# 

**Published:** 1868-01

**Authors:** 


### Books and Pamphlets Received.

A Practical Treatise on Surgical Apparatus, Appliances, and Elementary Operations; embracing Bandaging, Minor Surgery, Orthopraxy, and the treatment of fractures and dislocations. By Philip S. Wales, M. D., U. S. N. With six hundred and forty-two illustrations. Philadelphia: Henry C. Lea, 1867. For sale by Theodore Butler,

A Practical Treatise on the Diseases of Children. By D. Francis Condie, M. D., Fellow of the College of Physicians, etc., etc. Sixth edition, revised and enlarged. Philadelphia: Henry C. Lea, 1868. For sale by Theodore Butler.

On Diseases of the Lungs and Air Passages: their Pathology, Physical Diagnosis, Symptoms and Treatment. By Henry William Fuller, M. D., Fellow of the Royal College of Physicians, London, etc., etc. From the second and revised London edition. Philadelphia: Henry C. Lea, 1867. For sale by Theodore Butler.

Pennsylvania Hospital Reports, vol. 1., 1868- Philadelphia: Lindsay and Blakiston. For sale by Theodore Butler.

Obstetric Clinic: A practical contribution to the study of Obstetrics and the Diseases of Women and Children. By George T. Elliot, Jr., A. M., M. D., Professer of Obstetrics and Diseases of Women and Children, in the Bellevue Hospital Medical College, etc., etc. New York: D. Appleton & Co., 443, 445 Broadway, 1868. For sale by Martin Taylor.

Chronic Diseases of the Larynx, with special reference to Laryngoscopic Diagnosis and Local Therapeutics. By Dr. Albert Tobold, Lecturer to the University of Berlin. Translated from the German, and edited by George M. Beard, A. M., M. D., Lecturer on Nervous Diseases in the University of New York. Wm. Wood, & Co., 61 Walker street; 1868. For sale by Breed, Lent & Co.

Plastics: A new classification and a brief exposition of Plastic Surgery. A reprint from a report in the transactions of the Illinois State Medical Society for 1867. By David Prince, M. D. Philadelphia: Lindsay & Blackiston; 1868. For sale by Theodore Butler.

The Treatment of Diseases of the Throat and Lungs by Inhalations, with a new inhaling apparatus. By Emil Siegel, M. D. Translated from the second German edition by S. Nickles, M. D. Cincinnati: R. W. Carrol & Co., Pub., 1868. Max Mocher. Price $1.25.

Spermatorrhœa: Its Causes, Symptomology, Pathology, Diagnosis, Prognosis and Treatment. By Robert Bartholow, A. M., M. D., Professor of Materia Medica in the Medical College of Ohio, etc. Second Edition, Revised and Enlarged. New York: Wm. Wood & Co., 61 Walker Street; 1867. For sale by Breed, Lent & Co.

Ophthalmiastresche Beobachtungen von Dr. Med., Albert Mooren, Berlin, 1867. August Hirshwald. N. Shaws, 7 & 9 Brown street, New York.

Twelfth Annual Report of the Trustees of the State Lunatic Asylum at Northhampton; 1867.

Observations on the Nature and Treatment of Polypus of the Ear. By Edward H. Clark, M. D., Professor of Materia Medica, in the Harvard University, etc. Boston: Ticknor & Fields; 1867.

The Philosophy of Human Life, with especial design to develop the true Idea of Disease. Its nature, immediate occasion, and general remedy. By J. Jennings, M. D.

Pharmacy of the Cinchonas and of Podophyllum, with suggestions upon their therapeutic uses, modes of application, and tests of quality. By Edward R. Squibbs, M. D., Brooklyn, N. Y.

Seventeenth Anniversary Meeting of the Illinois State Medical Society, held in Springfield, June 4th and 5th, 1867.

Twenty-fourth Annual Report of the Managers of the State Lunatic Asylum, for the year 1866; transmitted to the Legislature February 5, 1867.

Union League Club of New York. Proceedings in reference to the Death of Gov. John A. Andrew, November 11, 1867.

Speech of Hon. Stephen I. Calahan, on the Admission to the Practice of Medicine and the Dispensing of Drugs.

Annual Report of the Surgeon-General United States Army, 1867.

Forty-second Annual Report of the Massachusetts Charitable Eye and Ear Infirmary, for the year ending September 30, 1867.

Annual Circular and Catalogue of the Long Island College Hospital, Brooklyn, N. Y.

How far do the Facts accompanying the Prevalence of Epidemic Cholera in Chicago, during the autumn of 1866, throw light on the Etiology of that Disease? By N. S. Davis, M. D.

